# Think-Aloud Testing of a Companion App for Colonoscopy Examinations: Usability Study

**DOI:** 10.2196/67043

**Published:** 2025-02-12

**Authors:** Christine Jacob, Roman Müller, Sonja Schüler, Alix Rey, Guillaume Rey, Berj Armenian, Alain Vonlaufen, Michael Drepper, Marius Zimmerli

**Affiliations:** 1 FHNW University of Applied Sciences and Arts Northwestern Switzerland, Riggenbachstrasse 16, Olten, 4600, Switzerland, 41 62 957 29 7; 2 Gimini Biosciences SàRL, Geneva, Switzerland; 3 International Institute for Management Development, Lausanne, Switzerland; 4 GGHA Geneva Gastroenterology & Hepatology Associates SA, Geneva, Switzerland; 5 Clarunis University Digestive Health Care Center, Basel, Switzerland

**Keywords:** eHealth, mobile health, mHealth, digital health, technology assessment, technology adoption, technology implementation, usability study, colonoscopy, app, application, examinations, smartphone, usability

## Abstract

**Background:**

Colonoscopies are vital for initial screening, follow-ups, surveillance of neoplasia, and assessing symptoms such as rectal bleeding. Successful colonoscopies require thorough colon preparation, but up to 25% fail due to poor preparation. This can lead to longer procedures, repeat colonoscopies, inconvenience, poorer health outcomes, and higher costs. eHealth tools can enhance bowel preparation and potentially reduce the need for repeat procedures.

**Objective:**

This usability study aimed to identify strengths and weaknesses in a prototype companion app for colonoscopy examinations. The objective was to obtain in-depth insights into the app’s usability, ease of use, and content comprehension, with the aim of refining the tool to effectively fulfill its intended purpose, guided by feedback from potential users.

**Methods:**

From February to August 2024, we conducted a qualitative study using the think-aloud procedure. Each session involved 6 tasks and a semistructured interview to delve deeper into participants’ task experiences. All think-aloud sessions and interviews were recorded. Quantitative usability questions were analyzed using Microsoft Excel, while qualitative data underwent coding and analysis based on thematic analysis principles.

**Results:**

In total, 17 individuals, all smartphone users, participated in this study. Participants were recruited from 1 hospital, 1 private clinic, and 1 patient organization in Switzerland. The study found that participants rated the app’s usability metrics positively, with an overall mean rating of ease of use at 4.29 (SD 0.59), usefulness at 4.53 (SD 0.72), and comprehensibility at 4.29 (SD 0.92). For the individual features, the mean ratings for ease of use were between 4 and 4.65, usefulness ranged from 4.35 to 4.82, and comprehensibility received ratings between 4.29 and 4.53, all measured on a 5-point scale, where 1 represented low agreement and 5 indicated high agreement. Additionally, 100% of participants indicated they will or may use the app if they require a colonoscopy examination. Participants highlighted the need for reminders and alerts in the week leading up to the colonoscopy, along with tailored content, simplified language, and visual aids.

**Conclusions:**

The app prototype demonstrated favorable results with the majority of participants, and the testing process enabled the prompt identification and resolution of usability issues. The next phase will prioritize and assess potential improvements based on urgency and feasibility to guide a focused development plan. Usability testing highlighted features such as push notifications and personalized content as top priorities for participants, making them key areas for immediate attention. Moving forward, the app has the potential to function effectively as a companion app for colonoscopy examinations. To achieve this, further studies with a larger sample in real-world settings will be crucial.

## Introduction

### Background

Colonoscopies are widely recognized as the most reliable method for detecting colorectal issues; their effectiveness and safety hinge on the thoroughness of bowel preparation beforehand [1,2]. Ensuring adequate bowel preparation is crucial for achieving clear visualization of the colon’s inner lining during the procedure. Poor bowel preparation is linked to risks such as missed significant lesions, procedural challenges, longer operation times, higher rates of interval colorectal cancers, and increased health care expenses [3]. However, a colonoscopy is an invasive procedure that demands extensive preparation. This includes taking a laxative, restricting food and liquid intake, and stopping certain medications in the week before the appointment.

Research indicates that up to 11% of individuals miss their colonoscopy appointments [4,5], and among those who do attend, many have insufficient bowel preparation, hindering clear colonic visualization [6-12]. A recent study investigated the efficacy of various bowel preparation regimens (4L, 2L, and ≤1L) for colorectal cancer screening, focusing on key quality indicators such as bowel cleanliness, cecal intubation rate, adenoma detection rate, and polyp detection rate, all aligned with the performance standards set by the European Society of Gastrointestinal Endoscopy (ESGE) [13]. While all regimens met the ESGE’s minimum quality thresholds, the adequacy of bowel preparation varied significantly between volumes [13]. Ultralow-volume preparations achieved an adequacy rate of 79%, notably lower than the 86.4% seen with high-volume preparations [13]. In particular, bowel preparation with sodium picosulfate and magnesium citrate (SPMC) and 1L polyethylene glycol with ascorbic acid (1L-PEGA) was adequate in only 75.2% and 82.9% of cases, respectively, highlighting the need for careful consideration when selecting a preparation method based on patient needs and procedural goals [13].

Misunderstanding dietary guidelines and cleansing instructions, along with noncompliance, significantly contributes to inadequate bowel preparation [14]. Ineffective bowel preparation can lead to several adverse outcomes, including reduced adenoma detection rates, extended procedure times, lower cecal intubation rates, increased electrocautery risks, and more frequent examination intervals [15,16]. To enhance patient adherence to colonoscopy procedures, various educational strategies have been used. Tools such as booklets, cartoons, and SMS text messaging have proven effective in increasing follow-up rates compared to standard care [17]. Furthermore, smartphone-based strategies have been developed to assist patients in preparing for colonoscopy [18-24]. Research indicates that these smartphone interventions generally lead to better outcomes, such as higher bowel cleansing quality scores, compared to usual care control groups [18-20,25,26]. However, there is limited evidence that these tools were designed with input from their intended users [27], which may reduce their effectiveness. Engaging potential users in the app development process is likely to enhance usability by ensuring the app’s content and features match user needs and preferences [28-30].

### Objectives

Considering that user research can significantly enhance a tool’s adoption and adherence rates post launch [28], this usability study was conducted to pinpoint strengths and weaknesses in the prototype of a companion app for colonoscopy examinations and to provide detailed insights into its quality regarding usefulness, ease of use, and content comprehension. The goal is to refine the product based on evidence gathered from potential users, ensuring it meets its intended purpose effectively.

## Methods

### App Prototype

The health care technology company Gimini Biosciences SàRL is developing digital health solutions to empower patients undergoing complex medical interventions. Their mission is to ensure every patient has access to the necessary information for a successful medical examination.

**Figure 1.**
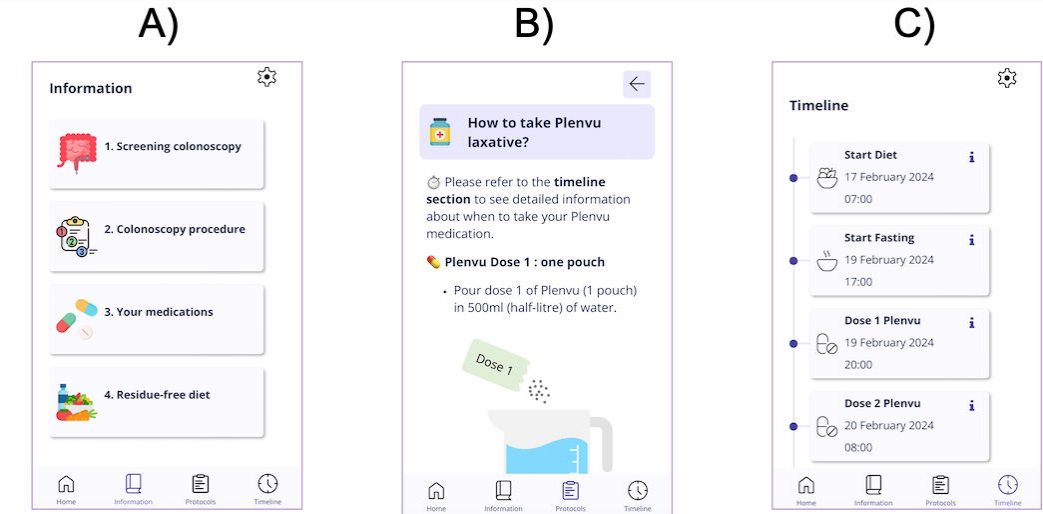
App prototype overview, including (A) patient information, (B) digital protocols, and (C) timeline and schedule.

The companion app for colonoscopy examinations, their first use case, features three main sections: (1) educational content about colonoscopy examinations; (2) digital protocols detailing diet, fasting, and laxative instructions; and (3) a personalized timeline based on the user’s examination date and time to guide them on diet, fasting, and laxative schedules. Figure 1 illustrates the design of the prototype, showcasing these sections.

### Study Design

The study involved conducting a qualitative interview with each participant using the standardized think-aloud method [31], which is described in detail below. Participants were guided using a semistructured test script to articulate their cognitive process in real time while completing a list of predefined tasks. Similar to many qualitative studies, this research used purposive sampling to gather in-depth insights [32]. Participants were selected based on their capacity to offer detailed and firsthand insights into the research topic, ensuring they could effectively articulate their real-life experiences [32,33].

### Participant Recruitment

The inclusion criteria covered individuals aged 40‐65 years who use a smartphone, have undergone a colonoscopy examination, have access to Wi-Fi and email, are comfortable using teleconferencing tools (eg, Microsoft Teams), and are capable of screen-sharing during the testing session. Three institutions played a key role in recruiting participants: Clarunis (the University Digestive Health Care Center of St. Clara Hospital and University Hospital Basel), GGHA (Geneva Gastroenterology & Hepatology Associates SA), and EUPATI Switzerland (European Patients’ Academy on Therapeutic Innovation).

Participants were directly approached and recruited by the collaborating institutions. Once a participant agreed to participate and signed the consent form, the respective institution forwarded the signed consent form along with the participant’s contact details to the core research team at the University of Applied Sciences and Arts Northwest Switzerland (FHNW). From that point, the research team took over the coordination and management of the participant’s involvement in the study.

Participants had the option to conduct the testing session in English, French, or German. The English version of the participant information sheet and consent form can be found in Multimedia Appendix 1. The information was also provided in both German and French, giving participants the freedom to select the language they felt most comfortable using, and the recruitment process spanned from February to July 2024. The researchers aimed to recruit a sufficient number of participants to achieve saturation, indicating that enough data had been collected when new information no longer provided additional insights [32,33].

### Think-Aloud Procedure

The think-aloud method is extensively used in app development as a popular tool for assessing usability [34]. In a think-aloud test, participants are asked to use the system and verbalize their thoughts continuously as they navigate through the user interface. This technique allows researchers to gain deeper insights into user misconceptions, which often lead to practical recommendations for redesign: when users misunderstand design elements, those elements may require modification. It also sheds light on why users make incorrect assumptions about certain aspects of the design and why they find other parts intuitive. The method is used to make cognitive processes, which would otherwise remain implicit and unspoken, more visible. By having participants verbalize their thoughts while performing a task, researchers can gain insights into thought processes, decision-making strategies, and individual patterns of interpretation.

Participants evaluated a web-based prototype of the app. They were instructed to explore the prototype while vocalizing their actions and observations, and to offer feedback on features, navigation, and perceived usefulness as they interacted with it. Researchers reminded participants to maintain a continuous stream of thoughts when needed and observed their behavior throughout the test tasks. All sessions were recorded, capturing participants’ screen interactions with the prototype. Detailed notes were taken during feedback sessions and stored using the research platform Tivian.

During each test session, participants were asked to complete 6 tasks, during which we observed their behavior and interactions with the tool. Additionally, they provided subjective assessments for each task, evaluating its usefulness, ease of use, and comprehensibility of the content. Textbox 1 displays the list of tasks participants were required to complete during the test sessions. The English version of the complete test script can be found in Multimedia Appendix 2.

Textbox 1. **The list of tasks participants were required to complete during the test sessions.**

Task 1: Customization and language choice

Task 2: First screen tour

Task 3: Accessing and navigating the background information section

Task 4: Accessing and navigating the fasting and food instructions

Task 5: Accessing and navigating the laxative instructions

Task 6: Accessing and navigating the meal and exercise recommendations

The think-aloud test was designed to encompass every possible task a user might perform within the app, covering all available functions and interactions. By including all tasks, we ensured a thorough capture of user feedback across the app’s entire functionality, providing a comprehensive assessment of the user experience. Below is a high-level summary of what each task involved:

- Task 1: Customization and language selection—In this task, participants were prompted to select their preferred language and adjust settings such as font size or content complexity, allowing them to personalize the app to their needs.
- Task 2: First screen tour—This task aimed to assess the intuitiveness of the app’s initial screen, encouraging participants to reflect on the clarity of the navigation and their understanding of the content available in each section.
- Task 3: Exploring background information—Here, participants were guided to access and navigate the section on background information about the colonoscopy examination, supporting their understanding of the procedure as they prepared for their appointment.
- Task 4: Reviewing fasting and dietary instructions—This task directed participants to find and review guidelines on what they can and cannot eat before the examination, including details on when to begin the special diet and the required fasting period.
- Task 5: Reviewing laxative instructions—Participants were asked to locate and understand instructions on taking the laxative, including information on dosage timing for effective preparation.
- Task 6: Reviewing meal and exercise recommendations—This task involved participants exploring the app’s suggestions for meals and exercises that could help them prepare optimally for the examination.

### Analysis

After compiling all notes from the testing sessions, CJ, RM, and SS collectively reviewed and synthesized the findings. These were then presented to the app development team for discussion, focusing on identifying features and functions requiring modification. The data encompassed audio recordings of the think-aloud sessions, observations noted by CJ, RM, and SS, and responses to usability questions.

Quantitative usability data, such as user ratings of ease of use and usefulness of the different features, were aggregated and analyzed using Microsoft Excel for Mac 2021 (version 16.86) to compute totals, percentages, means, and standard deviations. Qualitative participant comments were translated into English as needed for coding purposes. NVivo version 1.7.2 (QSR International), a qualitative data analysis software, was used for coding and categorizing the qualitative data. The data underwent thematic analysis to capture the depth and the unique interpretative contributions of individual researchers [35]. To ensure coding reliability, the first 3 authors, who conducted testing sessions in 3 different languages, engaged in collaborative discussions. The initial codebook was organized around core eHealth app development components: user interface (including navigation and visual design), user experience design (such as personalization), functionality (including notifications and reminders), and patient engagement and support (such as educational content). Initial coding was carried out by the first author (CJ), followed by a review from RM. Any coding discrepancies were addressed through discussions with SS until a consensus was reached.

### Ethical Considerations

The ethics committee of Northwest and Central Switzerland determined that ethics approval was not needed for this study, according to the Federal Act on Research Involving Human Beings, article 2, paragraph 1 (reference number Req-2023‐01506). All participants were briefed about the research background and signed a consent form agreeing to participate. Participants did not receive payment but were offered the opportunity for early and free access to the app upon its launch.

## Results

### Sample Characteristics

In total, 17 participants from 4 institutions tested the app prototype (3 of them were pilot tests, 1 in each of the 3 test languages). Participants were mostly male (13/17, 76%) and aged 40‐50 (8/17, 47%) years. The gender discrepancy among participants is largely due to recruitment challenges and the voluntary nature of the study. Since participation was optional, it may have led to a self-selection bias, where individuals more comfortable with technology or who have a specific interest in health applications were more likely to take part, resulting in a less balanced gender distribution among participants. Tests were conducted in German (6/17, 35%), French (6/17, 35%), and English (5/17, 29%). Table 1 presents the demographics and characteristics of the sample.

**Table 1.** Sample characteristics (N=17).

Characteristic		Values, n (%)
Gender		
	Male	13 (76)
	Female	4 (24)
Age (years)		
	<40	1 (6)^a^
	40‐50	8 (47)
	51‐60	5 (29)
	>60	3 (18)
Language		
	German	6 (35)
	French	6 (35)
	English	5 (29)
Referring institution		
	Clarunis^b^	9 (53)
	GGHA^c^	4 (24)
	FHNW^d^ (pilot testers)	3 (18)
	EUPATI Switzerland^e^	1 (6)

a One of the pilot tests, hence age inclusion criteria were not applied.

b Clarunis: The University Digestive Health Care Center of St. Clara Hospital and University Hospital Basel.

c GGHA: Geneva Gastroenterology & Hepatology Associates SA.

d FHNW: University of Applied Sciences and Arts, Northwestern Switzerland.

e EUPATI Switzerland: European Patients' Academy on Therapeutic Innovation, Switzerland.

### Usability Metrics

Usability metrics are specific measurements used to evaluate a digital product’s usability. These metrics typically assess how quickly users complete tasks, how often they make mistakes, and their overall satisfaction with the tool. By analyzing various usability metrics, we can gain a comprehensive understanding of the user’s experience and the tool’s overall usability.

During the testing sessions, we incorporated satisfaction metrics, which are subjective measures based on users’ self-assessments. These metrics evaluated the ease of use, usefulness, and content comprehensibility of different sections of the app, as well as their overall impression of the app. Responses were rated on a Likert scale from 1 (low agreement) to 5 (high agreement). Table 2 provides an overview of these subjective measures for the various tasks and the app as a whole. Some measures were not applicable for certain tasks. For example, in task 1 (customization and language selection), there was no text content to assess for comprehensibility, so this measure is marked as N/A (not applicable) for that task. In task 2 (first screen tour), participants were only asked to reflect on whether the initial screen was intuitive, rather than completing an action. Therefore, measures such as ease of use, usefulness, and comprehensibility were not rated for this task.

**Table 2.** Subjective usability measures (N=17).

Task	Ease of use^a^, mean (SD)	Usefulness^a^, mean (SD)	Comprehensibility^a^, mean (SD)
Task 1: Customization and language choice	4.41 (0.71)	4.35 (1.11)	N/A^b^
Task 2: First screen tour	N/A	N/A	N/A
Task 3: Accessing and navigating the background information section	4.41 (0.94)	4.76 (0.44)	4.53 (0.87)
Task 4: Accessing and navigating the fasting and food instructions	4 (1)	4.41 (0.87)	4.29 (0.99)
Task 5: Accessing and navigating the laxative instructions	4.53 (0.62)	4.82 (0.39)	4.35 (0.93)
Task 6: Accessing and navigating the meal and exercise recommendations	4.65 (0.7)	4.59 (0.62)	4.53 (0.8)
Overall satisfaction with the app as a whole	4.29 (0.59)	4.53 (0.72)	4.29 (0.92)

a User satisfaction of usability attributes was rated on a scale of 1 (low agreement) to 5 (high agreement).

b N/A: not applicable.

To provide a comprehensive assessment, test moderators also recorded observations on three additional usability metrics: (1) completion metrics (these measure whether users can successfully complete or partially complete tasks, indicating the tool’s effectiveness); (2) duration metrics (these track the average time users take to perform a task, reflecting the design’s complexity and the efficiency of user navigation); and (3) error metrics (these refer to actions users take that do not lead to the expected outcome, highlighting areas of confusion in the user interface or challenges with functionality). Table 3 presents an overview of these observed measures. Certain measures in this table were not applicable for task 2 (first screen tour), as participants were only asked to assess the intuitiveness of the initial screen rather than perform any specific action. As a result, metrics such as completion rate and error rate were not relevant for this task, since there was no actionable step for participants to complete or errors to quantify.

**Table 3.** Observed usability measures (N=17).

Task	Completion, n (%)	Error rate, n (%)	Duration (seconds), mean (SD)
Task 1: Customization and language choice	16 (94)	1 (6)	44.47 (32.39)
Task 2: First screen tour	N/A^a^	N/A	57.41 (45.94)
Task 3: Accessing and navigating the background information section	15 (88)	7 (41)	66.53 (46.32)
Task 4: Accessing and navigating the fasting and food instructions	16 (94)	7 (41)	75.41 (46.07)
Task 5: Accessing and navigating the laxative instructions	15 (88)	3 (18)	54.88 (47.89)
Task 6: Accessing and navigating the meal and exercise recommendations	17 (100)	3 (18)	56.76 (50.49)

a N/A: not applicable.

When asked if they would use the app for a colonoscopy examination once it becomes available, out of 17 participants, 14 (82%) said yes, 3 (18%) said maybe, and 0 (0%) said no.

### Qualitative Feedback

Participants provided comments and qualitative feedback on the 6 tasks they performed during the test sessions. While their feedback concerning the comprehensibility of the content, the user-friendliness, and the general user benefits of the application was mostly positive, they also expressed some confusion about certain content or features. Additionally, they offered suggestions for improvements in areas such as design and visualization as well as user guidance to address the gaps or issues they identified in the prototype. Overall, participants stressed the importance of receiving reminders and alerts in the week leading up to their colonoscopy. They also preferred tailored content, simplified language, and visual aids to enhance their understanding. Table 4 summarizes their qualitative feedback, organized into 4 key themes: app content, design, guidance, and features. These themes collectively illustrate the participants’ preferences and priorities, providing valuable insights for the development and improvement of the application.

**Table 4.** Key themes that emerged from the thematic analysis of the qualitative feedback.

Themes and subthemes		Qualitative feedback
Content		
	Content accuracy	Participants expressed a need for greater accuracy in the app’s content. Specifically, they suggested: Allowing the selection of examination times down to the minute, rather than in 15-minute intervals. Clearly stating that tea and hot drinks should not contain milk, as specified in the paper-based guidance. Ensuring the app accurately reflects the strict fasting phase, as some participants experienced longer fasting periods than indicated.
	Content clarity	Some content has been found to be unclear or confusing. For instance: The term “residue-free diet” may be difficult for laypeople to understand; “bowel cleansing” is suggested as a more straightforward alternative. The term “protocols” is ambiguous, and “preparation” is recommended as a clearer option. Instead of using the word “option” for meal examples, provide explicit examples to avoid misleading users into thinking these are the only choices available. For dosage instructions, emphasize that the liquid should be consumed in sips rather than all at once. The timeline entry “pickup medication” is unclear and potentially misleading; a more descriptive label is needed. More information on physical exercise should be included, such as its effects and importance in the preparation process.
	Content completeness	It has been noted that certain content is currently lacking in the app, namely: Expanded details on the colonoscopy procedure should be provided, with careful attention to wording. Starting the information section with cancer detection details can be perceived as alarming. It is essential to introduce the procedure in a reassuring manner, highlighting its benefits and emphasizing preventive care. Information on the effects of laxatives. Vegetarian menu options. Post-colonoscopy care and potential adverse events. Any assistance with laxative consumption would be greatly appreciated, as it can be quite unpleasant. For example, mixing the laxative with clear syrup to improve taste. Another tip is to sip water alternately with the laxative instead of consuming them sequentially, which can make the experience less difficult. Additional advice on making the process more manageable could include using a straw to drink the laxative or drinking tea beforehand to mitigate the salty taste.
	FAQs^a^	Participants have suggested including a section with FAQs to provide clear and concise answers to common concerns. Suggested questions include: Why is a colonoscopy performed? Include an explanation of its purpose and benefits, and note any differences in the procedure or considerations for men and women. Can I drive after taking the medication? When can I resume a normal diet? What are the advantages and disadvantages of various examination methods? Offer a comparison of different diagnostic options, such as stool examinations, to help users understand their relative benefits and limitations.
	Translation accuracy	A few minor translation inaccuracies have been identified: “Dose 1” was incorrectly translated into German; the correct term is “Dosis.” Under “fasting time,” “Untersuchung” is a more appropriate translation than “Prüfung.”
Design		
	Navigation clarity	Participants have proposed several improvements to enhance the clarity of the app’s navigation. Suggested enhancements include: Adjust the color scheme of the language selection navigation to make it more prominent. The button for confirming language selection should be available in multiple languages. Change the name of the navigation section labeled “Information” to better reflect that it pertains to the procedure rather than app-related information. The back arrow should be made more prominent by increasing its size and repositioning it centrally for better visibility and ease of use. There is uncertainty about the type of information the sections labeled “Protocols” and “Timeline” contain.
	Navigation structure	There were several suggestions to improve the app’s navigation structure: Increase the size of the customization icon and consider positioning it at the same level as the other main navigation icons to attract more attention. Keep content concise and allow users to click for more detailed information when needed. Display the timeline immediately after scheduling an appointment, as it is highly relevant. Ideally, place the timeline in the same section where laxative information is provided. Clearly mark the section for taking the laxative in the protocol and integrate the “how” with the “when” to provide a comprehensive guide. Distinguish and prioritize personalized content related to individual preparation for the colonoscopy from general informational content about the procedure, such as examination details and menu suggestions.
Guidance		
	Step-by-step guidance	Participants have reported some confusion and expressed a need for clearer guidance on navigating the app and understanding required actions. Specific recommendations include: Add a note indicating that users can change the language and the date of their intervention, as this option is not immediately apparent. Clearly explain why users need to enter their appointment details. Provide a clear explanation of the next steps immediately after entering appointment information, as some users were unsure where to click. Indicate that some content is customized based on the user’s appointment time to clarify how the app personalizes information. Include preliminary information about the general procedure at the start of the app, such as an overview page, to give users a better understanding of what to expect.
Features		
	Personalized notifications	Nearly all participants emphasized the critical need for personalized reminders and alerts, tailored to each patient based on the specific date and time of their colonoscopy appointment.
	Sharing and printing info	The importance of having the ability to print or share information directly from the app was emphasized.
	Tailored content	Participants noted that the app’s capability to customize content for individual users makes it more favorable compared to other methods of information delivery, such as paper. Specific suggestions include: Set the app to automatically select the language based on the user’s browser settings. Allow direct integration with the user’s calendar for streamlined timeline management. Ensure that the link directs users to the correct hospital right from the start. Add the medical center’s phone number or provide a direct link for users to easily contact the center.
	Visual aids	Participants recommended integrating visual aids to improve the clarity and comprehension of the information. Suggestions include: Add illustrations to assist in understanding the content more easily. Use visual cues to highlight the fasting time more prominently. Present information about meal preparation and dishes in a more visual format, including photos of menu items. Integrate photos and potentially a video to visually explain the procedure.

a FAQs: frequently asked questions.

## Discussion

### Principal Findings and Implications for App Improvement

Our findings indicate that participants were generally willing to use the companion app for colonoscopy examinations and found it mostly useful and easy to navigate. Usability is crucial for implementation, as technologies that are user-friendly are more likely to be consistently used over time. This is why leading technology acceptance frameworks such as the Technology Acceptance Model (TAM) and the Unified Theory of Acceptance and Use of Technology (UTAUT) emphasize ease of use and usefulness as key predictors for adopting new technologies [36,37]. The inclusion of subjective measures in assessments has been debated in the literature due to potential variability introduced by users’ subjective views. Despite this challenge, many scholars advocate for the inclusion of subjective criteria, such as ease of use and visual appeal, because they are fundamental drivers of adoption [38-41]. Therefore, incorporating subjective criteria, such as perceived ease of use, into the testing process could enhance tool adherence and improve health outcomes [42]. However, it is important to recognize that moderating factors such as a person’s age, education, and digital skills can influence their assessment of a technology’s perceived ease of use and overall usefulness [38].

Although participants rated the app highly for both ease of use and usefulness, they also offered several suggestions for improvement. For instance, they recognized that preparing for a colonoscopy involves multiple complex steps during the week leading up to the appointment. They expressed a desire for the app to send timely reminders to guide users throughout the process, aligning with findings from previous similar research [27]. Reminders are likely crucial for the effectiveness of the app. In fact, a randomized controlled trial found that without reminders the app group had the same quality of bowel cleanliness as the control group [21]. Previous studies have demonstrated that personalized reminders and alerts enhance the adoption of electronic health technologies, effectively modifying various health behaviors [43].

The app content was generally well received for its clarity and ease of understanding. However, there were a few requests for improved clarity or accuracy in certain areas, as detailed in the Results section. Additionally, users identified some content gaps in the tested prototype. Specifically, they requested more comprehensive guidance on how to take the laxative and minimize the unpleasantness of the experience. Furthermore, there was a need for information addressing post-colonoscopy issues. This latter finding aligns with similar research; for instance, Sewitch et al [27] also highlighted the perceived importance of post-colonoscopy information among potential users. Their study similarly underscored the need for detailed guidance on what to expect and how to manage any subsequent issues after the procedure. This is especially pertinent considering that statistics reveal approximately 25% of patients experience a minor adverse event within 48 hours of a colonoscopy, and 0.5% encounter a serious adverse event within 30 days [44].

Participants indicated a need for more detailed, step-by-step guidance on using the app. They sought clear instructions on the use of entering specific information, such as their procedure date and time, and how to effectively use the app to prepare for their examination. This feedback aligns with the extensive literature emphasizing the importance of user training and guidance to enhance adoption rates [38]. Introducing an app tour with guided notes detailing each section, coupled with clear instructions on features such as adjusting font size, could greatly enhance the overall user experience. This initial guidance would make it easier for users to understand how to navigate the app and maximize its use right from the start. Research suggests that users’ perception of ease of use can be significantly improved with high-quality training materials that guide them on how to effectively optimize these technologies [38,45,46].

**Figure 2.**
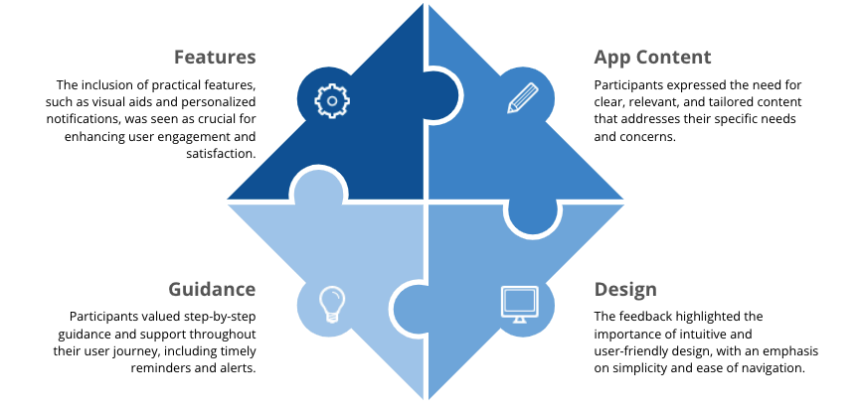
Key areas for app improvement.

Participants expressed a strong preference for more visual and interactive content, as well as a navigation design that prioritizes personalized information, such as the preparation timeline. They expressed a desire for information to be presented clearly at every stage of the colonoscopy preparation process. This feedback is consistent with previous research, which underscores the critical role of design elements and personalization in achieving successful patient adoption of digital health tools [38]. Several studies have specifically highlighted the importance of personalization. For example, a lack of customization options to meet individual needs can result in lower adoption rates or even abandonment of the tool [38,47]. Figure 2 provides a summary of our findings regarding user preferences for app design, content, features, and guidance. These insights will help boost the app’s usability and functionality, ultimately improving user adoption.

The next step will involve a thorough prioritization and impact assessment, where improvements according to urgency and feasibility will be ranked to guide a targeted development plan. For example, the usability testing results indicate that features such as push notifications and personalized content are top priorities for the participants. These will be addressed at the start of the next development cycle to ensure they are optimized before moving into real-world testing.

### Limitations and Future Research

While the practical insights from participants regarding the app prototype were valuable and guided us in making important modifications, it is important to acknowledge several limitations. Using the think-aloud methodology, there is a potential for reporting bias where participants may shape their responses to align with perceived researcher expectations; additionally, participants might selectively verbalize thoughts that align with what they believe interviewers want to hear, known as social desirability bias [48]. To address these potential biases, moderators were trained to actively encourage participants to provide verbal feedback and to collect observational data. These observations were used alongside participants’ subjective feedback during data analysis.

While the sample size of this study may appear small, previous research indicates that 80%‐90% of usability issues in websites and apps can be identified with 5 to 9 participants [49,50]. During the individual think-aloud sessions, recurring themes emerged, suggesting data saturation was achieved [32]. However, it is possible that additional participants could have uncovered different usability issues and provided diverse perspectives. Looking ahead, the app has promising potential as a companion for colonoscopy examinations. However, conducting larger-scale studies in real-world environments will be essential to validate and optimize its value and effectiveness.

### Conclusions

Overall, participants expressed satisfaction with the app’s usability. The think-aloud sessions provided real-time insights into the app’s appeal, relevance, and use. Minor adjustments to the prototype’s functionality were identified as necessary to enhance usability. Feedback and suggestions from participants have been integrated into the final app design. The initial findings from this usability study indicate that the app holds promising potential as a companion for colonoscopy examinations. This work establishes the foundation for further research to evaluate usability and feasibility among a larger, real-world population.

## Supplementary material

10.2196/67043 Multimedia Appendix 1 Participant information sheet and consent form.

10.2196/67043 Multimedia Appendix 2 Think-aloud usability test script.

## Abbreviations

1L-PEGA: 1L polyethylene glycol with ascorbic acid
Clarunis: the University Digestive Health Care Center of St. Clara Hospital and University Hospital Basel
ESGE: European Society of Gastrointestinal Endoscopy
EUPATI Switzerland: European Patients’ Academy on Therapeutic Innovation
FHNW: University of Applied Sciences and Arts Northwest Switzerland
GGHA: Geneva Gastroenterology & Hepatology Associates SA
SPMC: sodium picosulfate and magnesium citrate
TAM: Technology Acceptance Model
UTAUT: Unified Theory of Acceptance and Use of Technology
