# Supplementary material for: Think-Aloud Testing of a Companion App for Colonoscopy Examinations: Usability Study
Source: JMIR Hum Factors. 2025 Feb 12;12:e67043. doi: 10.2196/67043 (PMC11838146; doi:10.2196/67043)
Supplement: Multimedia Appendix 1 [file humanfactors-v12-e67043-s001.pdf]

Version 1.1 - Feb 2024

Participant Information Sheet

Usability Test of a Companion App for Colonoscopy Exams

Background: Failed medical interventions are a burden for healthcare systems. Importantly, patients are lacking the right support in terms of information to enable them to manage their preparation and recovery successfully.

Colonoscopy is a very common medical exam, used notably to screen for colon cancer. Studies show that 1 in 5 patients are not well prepared for this type of exam. Failed colonoscopy exams have important medical consequences, as it leads to undiagnosed colorectal cancer patients, and a waste of healthcare resources.

The healthcare technology provider Gimini is building digital health solutions empowering patients for medical interventions. The goal is to help every patient to have access to the information they need to ensure a successful medical exam.

Objective: Gimini created its first prototype for lower endoscopy examinations and asked the University of Applied Sciences Northwestern Switzerland (FHNW) to help them test it with potential users. The study would enable them to validate the prototype with potential users, to ensure acceptance and improve the app based on their feedback.

The test will be done in the form of individual online user testing (according to the participant availability) to assess:

- **Usability:** better understand how users interact with the app prototype and identify potential issues that may reduce patient engagement.
- **Customization**: assess the user acceptance of customization features including language, font size and content complexity.
- **Readability:** assess the prototype's ability to provide easily understandable information to users to truly help them manage their medical interventions.

Expected outcomes: the prototype testing will help identify potential weaknesses in the prototype. It will also provide detailed information on its quality, in terms of usability to improve patient compliance, and assessing the content comprehension. It will help the app provider in developing a product that can fulfil the desired role and improve it based on the feedback collected from users.

Funding: This study is sponsored by Innosuisse (the Swiss innovation agency), grant number: 70325.1 INNO-ICT.

n|w Fachhochschule Nordwestschweiz gimini

Participant Information FAQs

1. **Who can participate?** Individuals aged between 40-65 years that use a smartphone, had a lower endoscopy exam, have access to wifi and email, and are comfortable using Teleconferencing tools (e.g. MS Teams) and capable of screen-sharing during the testing session.

2. You can **refuse to take part** in this study without giving a reason.

3. **Ethical approval:** The Ethics Committee of Northwest and Central Switzerland (EKNZ) determined that ethical approval was not required for this study according to the Federal Act on Research involving Human Beings, article 2 paragraph 1 (reference number Req-2023-01506).

4. **What will happen to the results of the study?** The study results will be used to optimise the app and may be published in academic journals and presented at conferences.

5. **What will I be asked to do?** Based on their preference and availability, users can participate through a 30-40 min individual online testing session where you will be asked to share your screen and perform specific tasks on the app prototype and give your feedback.

6. **Your participation in the study will be anonymised.** Only the research team will have access to participants' data. No personal or identifiable data be included in the dissemination of the results, which will be anonymised.

7. **Information that is collected from you** will be securely held. Personal identifiable information (e.g. consent forms) will be kept separately from the data. Participants will be assigned a study code number and identifying information stored separately from the data.

8. **Recording equipment** will be used to record the testing session.

9. **You can withdraw from the study without** giving a reason. To do so please e-mail christine.jacob@fhnw.ch. The last time it will be possible to withdraw your data is the end of April 2024, given it will not be possible to withdraw once the research analysis and write up has started or findings were published.

10. You do not have to answer any questions you do not wish to.

11. **Free app access:** the research budget won't allow for participant remuneration; however, the research team foresees a relevant social value in enhancing adherence and consequently intervention success once the app is launched. **Participants to the prototype testing will have early and free access to the app once it's launched (*email required*).**

12. If you have **any questions or complaints** about the study, please contact the main investigator at christine.jacob@fhnw.ch.

Version 1.1 - Feb 2024

Participant Consent Form

Title of the study: *Usability Test of a Companion App for Colonoscopy Exams*

Main investigator and contact details: Dr. Christine Jacob christine.jacob@fhnw.ch

1. I agree to take part in the above research. I have read the Participant Information Sheet (Version 1.1 - Feb 2024) for the study. I understand what my role will be in this research, and all my questions have been answered to my satisfaction.
2. I understand that I am free to withdraw from the research at any time until the end of April 2024, without giving a reason.
3. I am free to ask any questions at any time before and during the study.
4. I understand what will happen to the data collected from me for the research.
5. I have been provided with a copy of the Participant Information Sheet Version 1.1 - Feb 2024.
6. I understand that non-identifiable quotes from me could be used in the dissemination of the research, and I am expressing my personal views.
7. I understand that the testing session will be recorded.

Data Protection and ethical approval: I agree to the University[1] processing personal data, which I have supplied. I agree to the processing of such data for any purposes connected with the Research Project as outlined to me*. Recordings will be retained for a period of 12 months and deleted afterwards.

The Ethics Committee of Northwest and Central Switzerland (EKNZ) determined that ethical approval was not needed for this study according to the Federal Act on Research involving Human Beings, article 2 paragraph 1 (reference number Req-2023-01506).

Name of participant ……………………………

Email ……………………………

Phone number ……………………………

Signature / Date ……………………………

I WISH TO WITHDRAW FROM THIS STUDY.

If you wish to withdraw from the research, please speak to the researcher or email them at christine.jacob@fhnw.ch stating the title of the research. You do not have to give a reason for why you would like to withdraw. Please let the researcher know whether you are/are not happy for them to use any data from you collected to date in the write up and dissemination of the research.

Participant Identification Number for this study: ……………………………….

[1] "The University" refers to the University of Applied Sciences Northwestern Switzerland (FHNW)
