# Supplementary material for: Think-Aloud Testing of a Companion App for Colonoscopy Examinations: Usability Study
Source: JMIR Hum Factors. 2025 Feb 12;12:e67043. doi: 10.2196/67043 (PMC11838146; doi:10.2196/67043)
Supplement: Multimedia Appendix 2 [file humanfactors-v12-e67043-s002.pdf]

Fachhochschule Nordwestschweiz gimini

Usability Test Script

Usability Test of a Companion App for Colonoscopy Exams

Best practices for test moderators:

- Treat participants with respect and make them feel comfortable.
- Remember that you are testing the app, not the users. Help them understand that they are helping us test the prototype.
- Remain neutral – you are there to listen and watch. If the participant asks a question, reply with “What do you think?” or “I am interested in what you would do.”
- Do not jump in and help participants immediately and do not lead the participant. If the participant gives up and asks for help, you must decide whether to end the scenario, give a hint, or give more substantial help.
- Take good notes. You should capture what the participant did in as much detail as possible as well as what they say (in their words). The better the notes that are taken during the session, the more meaningful the analysis will be.
- Measure both performance and subjective (preference) metrics. People's performance and preference do not always match. Often users will perform poorly but their subjective ratings are very high. Conversely, they may perform well but subjective ratings are very low.
    - Performance measures include: success, time, errors, etc.
    - Subjective measures include: user's self reported satisfaction and comfort ratings.

Usability metrics definition (for moderators):

- ***Scenario completion:*** The scenario is completed when the participant indicates the goal has been obtained (whether successfully or unsuccessfully) or the participant requests guidance
- ***Time on task:*** time to complete each requested task
- ***Error rate:*** e.g. initially selecting the wrong function, using a user-interface control incorrectly such as attempting to edit an uneditable field
- ***Subjective satisfaction:*** the user’s subjective assessment of ease of use and satisfaction
- ***Readability:*** if applicable, the user’s subjective assessment of understandability and clarity of the content

Explain the aim and benefits of the usability test to the participant

Hello my name is ……., I work for the FHNW, and we are doing this research on behalf of Gimini, a healthcare technology provider developing companion apps that aim to help and support people with information and step-by-step guidance for their medical exams to enhance adherence and success rates of medical examinations that require relatively complex or multi-step preparation.

We this test we aim to:

- Learn if participants are able to complete specified tasks successfully (this is to test the ease of use of the tool not the capabilities of the user)
- Identify how long it takes to complete specified tasks
- Find out how satisfied participants are with the app
- Identify changes required to improve user performance and satisfaction

The facilitator will instruct the participant to ‘think aloud’ so that a verbal record exists of their interaction with the tool.

Emphasise the following:

- We want to see if our app works well for users. It should take about 30-40 minutes
- **We are testing the app, and not you!** Don’t worry if you make any mistakes, we’re here to learn from your experience
- You won’t hurt our feelings, our aim is to improve the app
- Please try to **think out loud** as you do the tasks
- Ask me if you have questions, but understand that I may not answer them immediately because we’re trying to see how you would complete these tasks if you were on your own
- We are recording the session. Only the research team will access this recording.

NOTE FOR MODERATOR: START RECORDING

Demographic info:

- Gender: , M , F
- Age: , 40-50 , 51-60 , above 60

Task 1 - Customization of the app and language choice

First, I’m going to ask you to open your web browser and share your screen with me.
Once it is working properly, I’d like to ask you to open up the weblink I am sending you in the chat so we can start the app prototype testing
(link → app.gimini.ch/?pathway=Cohort1_PlenvuSplitDose_)

Could you please go to the app section where you can change the language and select your language of choice? (e.g. DE/FR)

You could also customise the font size or content complexity if you wish to do so.

Performance Measures (moderator notes)

- Task completion , task completed , partially completed , not completed
- Error rate , no error , error (please describe)

Subjective Measures (participant rating)
On a scale of 1 to 5 (with 1 completely disagree and 5 completely agree) please rate:

- The task was easy to complete ,1 ,2 ,3 ,4 ,5
- The feature is useful ,1 ,2 ,3 ,4 ,5

Is there anything missing in this feature/section, or something we should change to make this easier and or more useful to you?

Task 2 - First screen tour

Now, let's save your changes and go back to the start screen… before you start doing anything, just look at the screen and tell me what you make of it: what you think you can do with it, and what it's for.

Just look around but please don't "click" (or tap) on anything yet.

Is the navigation clear?

Note to moderators: please take notes of how clear the different sections are to the participant (i.e. are the section names/icons self explanatory) and whether they understand their utility (i.e. whether they understand what each section is for and what the app generally does)

Task 3 - Accessing and navigating the background information section

Imagine that you just scheduled a lower endoscopy exam with your care team and you want to learn more about the process. Could you please go to the app section that holds this information and navigate through it?

Performance Measures (moderator notes)
- Task completion task completed partially completed not completed
- Error rate no error error (please describe)

Subjective Measures (participant rating)
On a scale of 1 to 5 (with 1 completely disagree and 5 completely agree) please rate:
- The task was easy to complete 1 2 3 4 5
- The content is useful 1 2 3 4 5
- The content is clear and easy to understand 1 2 3 4 5

Is there anything missing in this feature/section, or something we should change to make this easier and or more useful to you?

Task 4 - fasting and food instructions

Imagine that your lower endoscopy exam is approaching and you want to inform yourself and follow the strict instructions for the preparation.

Could you find out about the foods you are allowed or not allowed to eat before the exam?
Try to find out if you are allowed to drink water during the fasting period before the exam.

Could you please look up the information about when to start the special diet for the exam?
Could you please look up the information about when to start fasting for the exam?
Could you please look up the information about when to start the strict fasting for the exam?

Performance Measures (moderator notes)
- Task completion task completed partially completed not completed

- Error rate ○ no error ○ error (please describe)

Subjective Measures (participant rating)
On a scale of 1 to 5 (with 1 completely disagree and 5 completely agree) please rate:
- The task was easy to complete ○1 ○2 ○3 ○4 ○5
- The content is useful ○1 ○2 ○3 ○4 ○5
- The content is clear and easy to understand ○1 ○2 ○3 ○4 ○5

Is there anything missing in this feature/section, or something we should change to make this easier and or more useful to you?

Task 5 - laxative instructions

Could you please inform yourself about how to take the laxative?
Could you please look up the information about when to take each dose of the laxative?

Performance Measures (moderator notes)
- Task completion ○ task completed ○ partially completed ○ not completed
- Error rate ○ no error ○ error (please describe)

Subjective Measures (participant rating)
On a scale of 1 to 5 (with 1 completely disagree and 5 completely agree) please rate:
- The task was easy to complete ○1 ○2 ○3 ○4 ○5
- The content is useful ○1 ○2 ○3 ○4 ○5
- The content is clear and easy to understand ○1 ○2 ○3 ○4 ○5

Is there anything missing in this feature/section, or something we should change to make this easier and or more useful to you?

Task 6 - meal & exercise recommendations

Could you please check some meal suggestions?
Now, could you also please check how exercise can help prepare for the exam?

Performance Measures (moderator notes)
- Task completion ○ task completed ○ partially completed ○ not completed
- Error rate ○ no error ○ error (please describe)

Subjective Measures (participant rating)
On a scale of 1 to 5 (with 1 completely disagree and 5 completely agree) please rate:
- The task was easy to complete ○1 ○2 ○3 ○4 ○5
- The content is useful ○1 ○2 ○3 ○4 ○5
- The content is clear and easy to understand ○1 ○2 ○3 ○4 ○5

Is there anything missing in this feature/section, or something we should change to make this easier and or more useful to you?

n|w Fachhochschule Nordwestschweiz gimini

Overall User Experience (Subjective Measures)

We are almost done with our testing, and to close, we want you to rate your overall experience with the app as a whole.

On a scale of 1 to 5 (with 1 completely disagree and 5 completely agree) please rate:

- The app is generally easy to use 1 2 3 4 5
- The app would be useful in preparing me for a lower endoscopy 1 2 3 4 5
- The app content is generally clear and easy to understand 1 2 3 4 5
- If available, I would use the app if I need a lower endoscopy exam yes no maybe

(Note to Moderators: prompt questions - if they disagree with the above, prompt with questions such as: so why would you not choose to use the app? What features or changes would motivate you to use it? Make it more useful…etc)

Is there anything missing in the app in general, or something we should change to make the app easier to use and / or more useful to you?

As a participant in this testing you qualify for early and free access to the app once it's launched. If you wish to have it, please provide your email so we can send you the link once the app is ready (*sharing your email indicates your consent to receiving an email with the app information and link from Gimini when it is ready*).

Participant email in case of desire to get free access to the app: *(as per consent form?)*
